# Optimization of Recycled a-FePO_4_/rGO Composites via Thermal Reduction for Enhanced-Performance Lithium-Ion Batteries

**DOI:** 10.3390/ma18214850

**Published:** 2025-10-23

**Authors:** Shuchun Hu, Jinde Yu, Hua Chen, Zengbin Lin, Fengchun Zhang, Meiling Guo, Aipeng Zhu, Yin Liu

**Affiliations:** 1 Yibin Research Institute & School of Materials and Environmental Engineering, Chengdu Technological University, Chengdu 611730, China; schu@home.swjtu.edu.cn (S.H.); zfchun@cdtu.edu.cn (F.Z.); guomeiling0913@163.com (M.G.); zhuaipeng@cdtu.edu.cn (A.Z.); liuyin9114@163.com (Y.L.); 2 Sichuan Contemporary Amperex Technology Co., Ltd., Yibin 644005, China; chenh39@catl.com; 3 Contemporary Amperex Technology Co., Ltd. (CATL), Ningde 352100, China; linzb01@catl.com

**Keywords:** lithium-ion batteries, spent LiFePO_4_ recycling, amorphous FePO_4_/rGO composites

## Abstract

In this study, spent LiFePO_4_ (LFP) cathode materials were recycled and transformed into amorphous FePO_4_ (a-FePO_4_) via a leaching–precipitation method for further use as a high-performance cathode material in lithium-ion batteries. To enhance its electrochemical performance, a-FePO4 was composited with graphene oxide (GO) and subsequently reduced at different temperatures (250–450 °C) under N_2_ atmosphere to obtain a series of a-FePO_4_/rGO composites. Among them, the a-FePO_4_-rGO-1 (reduced at 250 °C) exhibited the most superior performance, delivering a specific high discharge capacity of 117 mAh·g^−1^ after 100 cycles at 1 C and an excellent rate capability of 98 mAh·g^−1^ even at 10 C. Electrochemical impedance spectroscopy revealed that this composite possessed the lowest charge transfer resistance and most efficient Li^+^ diffusion kinetics. This study demonstrates that moderate-temperature thermal reduction is a critical strategy for optimizing the conductive network of a-FePO_4_-rGO composites, thereby significantly improving the electrochemical properties of recycled FePO_4_-based cathode materials.

## 1. Introduction

The rapid expansion of the electric vehicle and portable electronics market has led to a massive deployment and subsequent decommissioning of lithium-ion batteries (LIBs), giving rise to serious concerns regarding resource sustainability and environmental protection [1,2,3]. Among various cathode materials, lithium iron phosphate (LiFePO_4_, LFP) is widely used in commercial applications due to its notable advantages, including high thermal stability, long cycle life, and low toxicity. Consequently, developing efficient and eco-friendly strategies for recycling valuable components from spent LFP batteries has become both an economic necessity and an environmental imperative [4,5,6,7,8].

Iron phosphate (FePO_4_) [8,9], a key precursor for the synthesis of LFP, stands as a primary target for recovery from end-of-life batteries. Conventional recycling processes often yield FePO_4_ in its crystalline quartz-type phase. While thermodynamically stable, it suffers from intrinsic limitations such as low electronic and ionic conductivity, which restrict its electrochemical performance. In contrast, amorphous FePO_4_ (a-FePO_4_) has emerged as a promising alternative cathode material owing to its open framework structure, shortened ion diffusion pathways, and larger specific surface area. These characteristics contribute to improved lithium-ion intercalation/deintercalation kinetics and enhanced reversible capacity [10,11]. Nevertheless, the practical deployment of a-FePO_4_ remains challenging due to its poor electrical conductivity, which leads to unsatisfactory rate capability and cycling stability.

To overcome this limitation, compositing with conductive carbon materials [12,13,14], particularly graphene oxide (GO) and reduced graphene oxide (rGO), has proven effective [15,16,17,18,19]. The two-dimensional structure and high electrical conductivity of rGO can form an efficient percolating network within the composite. Moreover, the in situ strategy employed in this work is widely reported to yield superior interfacial contact and synergistic effects, which further facilitate electron transport and accommodate volume changes during cycling [20,21]. The reduction process from GO to rGO, typically achieved through thermal or chemical treatments, is critical as it directly influences the electrical properties and interfacial characteristics of the final composite material. However, this process must be carefully optimized: excessively high reduction temperatures may not only degrade the structural integrity of the carbon framework but could also induce crystallization of the a-FePO_4_, thereby undermining its electrochemical advantages [22]. Recent studies have demonstrated that the thermal reduction in GO significantly alters its electrical conductivity, dielectric behavior, and optical constants, particularly in the range of 250–450 °C. For example, Politano and Versace [23] reported that the electrical resistance of GO films decreases by nearly two orders of magnitude upon annealing at 450 °C, accompanied by a marked increase in optical conductivity. Similarly, Amiri et al. [24] performed high-precision correlative analyses revealing that dielectric anisotropy and permittivity of GO films evolve strongly with annealing parameters, emphasizing the importance of controlled thermal treatment in tuning electronic properties. These studies provide valuable insights and guidance for our work, particularly in balancing the conductivity enhancement of rGO with the structural stability of amorphous FePO_4_, thereby offering a meaningful direction for optimizing the overall electrochemical performance of the recycled composite cathode.

In this study, we demonstrate a sustainable and controllable route for fabricating high-performance a-FePO_4_-rGO composite cathodes using spent LFP as the raw material. Quartz-type FePO_4_ was recovered from spent batteries via acid leaching, followed by its transformation into a-FePO_4_ through a leaching–precipitation method. The a-FePO_4_ was then integrated with GO and subjected to controlled thermal reduction at various temperatures (250–450 °C). The correlation between the reduction in temperature and the resulting structural, morphological, and electrochemical properties was systematically investigated.

This study distinguishes itself through a novel in situ reduction and compositing method that overcomes the poor conductivity of recycled FePO_4_. Our work further provides fundamental insights into how thermal reduction tunes the interface and conductivity, outlining a practical pathway for recycling spent LFP batteries into high-performance cathodes.

## 2. Materials and Methods

### 2.1. Synthesis of Recycled FePO_4_

In total, 1 g of spent LFP powder (recycle) was mixed thoroughly with 1 M H_2_SO_4_ (AR, Chengdu, China) in a round-bottom flask. The slurry was kept in a 60 °C water bath and 30 wt % H_2_O_2_ was added dropwise under stirring. After 1.5 h of reaction (molar ratio LFP:H_2_SO_4_:H_2_O_2_ = 1:0.65:2.8), the mixture was filtered, and the residue was dried at 80 °C for 3 h. The dried solid was then calcined in a tube furnace at 600 °C in an air atmosphere for 4 h to yield recycled FePO_4_ [25].

### 2.2. Synthesis of GO Dispersion Solution

Under ice-bath cooling (0–2 °C), 23 mL of 98% H_2_SO_4_ was slowly added to a 250 mL Erlenmeyer flask placed on a magnetic stirrer. After sequential addition of 1 g graphite and 0.5 g NaNO_3_ (AR, Chengdu, China), the mixture was stirred for 3 min. 3 g KMnO_4_ (AR, Chengdu, China) was then introduced in three portions while maintaining the internal temperature below 20 °C. After reacting for 2 h, the ice bath was removed, and the temperature was raised to 35 °C with continued stirring for 30 min. Deionized water (46 mL, 15 °C) was added, raising the temperature to 98 °C and continuous heating for 20 min until the solution turned brownish-yellow and emits red smoke. Subsequently, 5 mL of 30% H_2_O_2_ was added to the reaction. The product was separated by repeated centrifugation/washing cycles until no white precipitate was detected with BaCl_2_ (AR, Chengdu, China), and the resulting solid was dried at 60 °C under vacuum for 12 h to yield brown oxidized graphite. Finally, 0.10 g of the as-obtained solid was dispersed in 50 mL of deionized water, stirred for 3 min, and sonicated at ambient temperature for 10 min to afford a homogeneous GO dispersion with a concentration of 2 mg/mL [26].

### 2.3. Synthesis of a-FePO_4_

In total, 2 g of the recycled FePO_4_ were transferred into a 50 mL round-bottom flask, followed by the addition of 20 mL of a mixed acid solution (1.5 M HNO_3_ and 1.5 M H_3_PO_4_, AR, Chengdu, China). The suspension was heated at 90 °C for 4 h under magnetic stirring. After filtration, the obtained leachate was transferred to a beaker, and its pH was adjusted to 2 with aqueous NH_3_. The precipitate was collected by filtration, washed thoroughly with deionized water, and vacuum-dried at 80 °C for 12 h to afford amorphous FePO_4_ (denoted as a-FePO_4_).

### 2.4. Synthesis of a-FePO_4_-GO and Its Derivatives

In total, 20 mL of the above leachate was mixed with 20 mL of a 2 mg/mL GO aqueous dispersion. The pH of the mixture was adjusted to 2 with NH_3_, and the resulting suspension was transferred into a 100 mL Teflon-lined autoclave. Hydrothermal treatment was conducted at 80 °C for 12 h. After cooling, the black precipitate was separated by centrifugation, washed repeatedly with deionized water and ethanol, and vacuum-dried at 80 °C for 12 h to give a-FePO_4_-GO. The as-prepared a-FePO_4_-GO was placed in a vacuum tube furnace and heated at 2 °C min^−1^ to 250, 350 or 450 °C under N_2_ atmosphere, held at the target temperature for 6 h to reduce GO to rGO [27]. The corresponding products are designated as a-FePO_4_-rGO-1, a-FePO_4_-rGO-2 and a-FePO_4_-rGO-3, respectively.

### 2.5. Battery Assembly and Material Characterization

The assembly of CR2025 coin cells was conducted in an argon-filled glovebox (H_2_O/O_2_ < 1 ppm). Each cell was constructed by sequentially layering the following components: negative can, electrolyte (2–3 drops), working electrode, separator, additional electrolyte (5–7 drops), Li counter electrode, spacers, spring, and positive can. The cells were crimp-sealed under pressure and allowed to rest for 12 h prior to electrochemical testing. Microstructural analysis of a-FePO_4_ and its composite materials was carried out using a Thermo Scientific Apreo 2C scanning electron microscope (Thermo Fisher Scientific, Waltham, MA, USA) with an accelerating voltage of 20 kV. X-ray diffraction (XRD) patterns were acquired on a Rigaku Ultima IV multipurpose X-ray diffractometer (Rigaku Corporation, Tokyo, Japan) employing Cu-Kα radiation (λ = 1.5406 Å) operated at 40 kV and 30 mA. Data were collected over a 2θ range from 10° to 80° with a step size of 0.02°. X-ray photoelectron spectroscopy (XPS) was performed on a Thermo Scientific K-Alpha spectrometer (Thermo Fisher Scientific, USA) using a monochromatic Al-Kα X-ray source (1486.6 eV) at 12 kV and 6 mA. High-resolution spectra were recorded to determine elemental composition and valence states.

### 2.6. Electrochemical Measurements

Charge/discharge tests were performed on a CHI-7660E electrochemical workstation in a three-electrode setup, with a-FePO_4_-based composites as the working electrode and lithium foil serving as both counter and reference electrodes. Electrochemical impedance spectroscopy (EIS) was implemented on the same CHI-7660E system, covering a frequency spectrum from 0.01 Hz to 1 MHz with an AC perturbation amplitude of 0 mV. All electrochemical data were collected from a minimum of four coin-cells per sample, with the values presented being the corresponding averages. The variation among different batches was less than ±5%, indicating excellent reproducibility of the recycled a-FePO_4_-based electrodes.

## 3. Results

### 3.1. Structural and Morphological Characterization of the Materials

**Figure 1:**
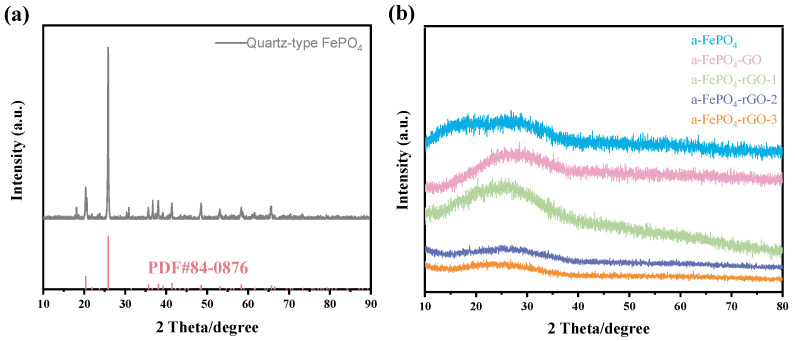
XRD patterns of FePO_4_ and its composites prepared using different methods. (**a**) quartz-type FePO_4_; (**b**) a-FePO_4_ and its composites treated by different calcination temperatures.

XRD is a powerful technique for characterizing the structure and phase composition of materials. Figure 1 shows the XRD patterns of FePO_4_ and its composites prepared by different methods. In Figure 1a, the XRD pattern of recycled FePO_4_ obtained through hydrometallurgy exhibits characteristic peaks that correspond well to the standard card #84-0876, confirming the successful obtaining of quartz-type FePO_4_. Figure 1b displays the XRD patterns of FePO_4_ synthesized via the leaching–precipitation method and its corresponding composites. It can be observed that all five products show no sharp or intense diffraction peaks, but only a broad amorphous hump near 25°, indicating typical short-range order characteristics of an amorphous material. This confirms that the FePO_4_ in these modified products exists in an amorphous state. Therefore, the leaching–precipitation method successfully achieved the transformation from quartz-type FePO_4_ to amorphous FePO_4_ (a-FePO_4_). Furthermore, the incorporation of GO and subsequent high-temperature treatment did not alter the amorphous nature of FePO_4_, as all composites remained amorphous. It is worth noting that with increasing temperature, the intensity of the broad amorphous background in the XRD patterns gradually decreases from a-FePO_4_-GO to a-FePO_4_-rGO-3, suggesting that the a-FePO_4_ may be undergoing a gradual transition toward a crystalline phase. However, even when the calcination temperature increased to 450 °C, a-FePO_4_-rGO-3 still showed no distinct diffraction peaks and remained amorphous. These results indicate that the amorphous structure of the composites can be preserved when the reduced temperature is controlled at or below 450 °C. Overall, the XRD analysis clearly demonstrates the successful amorphization of FePO_4_ and the relative structural stability of its composites under various processing conditions.

**Figure 2:**
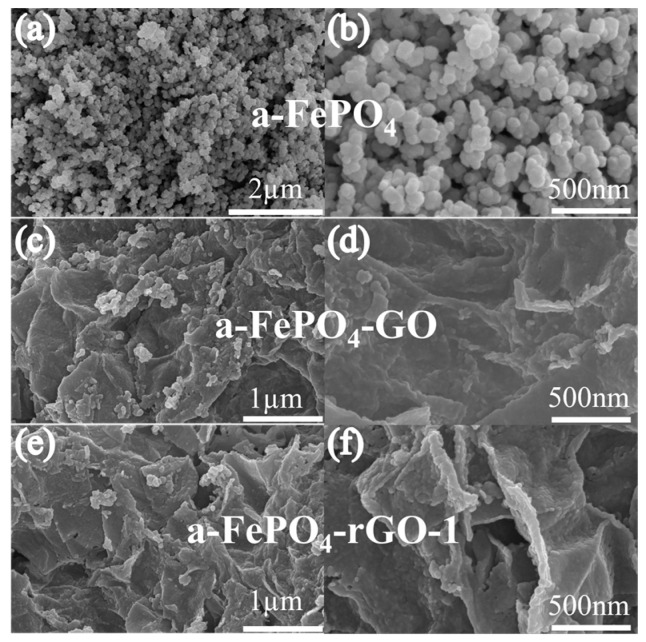
The SEM images and corresponding magnified views of a-FePO_4_ and its composites: (**a**,**b**) a-FePO_4_; (**c**,**d**) a-FePO_4_-GO; (**e**,**f**) a-FePO_4_-rGO-1.

Figure 2 shows the scanning electron microscope (SEM) images of a-FePO_4_, a-FePO_4_-GO, and a-FePO_4_-rGO-1. As can be seen in Figure 2a,b, the a-FePO_4_ sample without GO exhibits a porous spherical morphology with particle sizes ranging from 30 to 50 nm, which is governed by the principle of minimal free energy. Figure 2c,d reveal that the particle size of a-FePO_4_-GO is smaller than that of pure a-FePO_4_. This reduction is due to the incorporation of GO, which forms a two-dimensional layered structure and provides a high specific surface area for a-FePO_4_. Moreover, the active oxygen-containing functional groups on the GO surface facilitate the uniform distribution of a-FePO_4_ nanoparticles, thereby inhibiting their agglomeration and leading to the formation of a well-dispersed a-FePO_4_-GO composite. Although the a-FePO_4_-rGO-1 composite (Figure 2e,f) may undergo the transformation from GO to rGO during high-temperature reduction, no significant difference in composite morphology was observed compared with a-FePO_4_-GO. Additionally, as shown in Figure S1, both a-FePO_4_-rGO-2 and a-FePO_4_-rGO-3 display a three-dimensional porous microstructure, with particle sizes also in the range of 30–50 nm. However, under calcination, the thermal decomposition of the precursor a-FePO_4_-GO enhances the surface activity of particles on the GO sheets, promoting particle diffusion and interaction with adjacent particles, which ultimately leads to the agglomeration of a-FePO_4_ particles.

**Figure 3:**
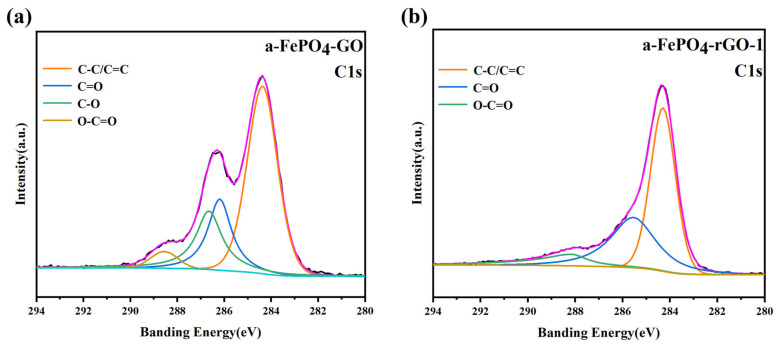
High-resolution XPS C1s spectra of (**a**) a-FePO4-GO; (**b**) a-FePO4-rGO-1.

XPS is widely employed to analyze the surface elemental composition and chemical states of materials. Figure S2a–c shows the XPS survey spectra of a-FePO_4_ and its composites with GO, confirming the presence of Fe, P, O, and C in all samples. Figure 3a,b display the high-resolution C 1s spectra of a-FePO_4_-GO and a-FePO_4_-rGO-1. After peak deconvolution, the C 1s spectra of a-FePO_4_-GO (Figure 3a) exhibits four peaks at binding energies of 284.8 eV, 286.3 eV, 286.9 eV, and 288.3 eV, corresponding to C–C/C=C, C=O, C–O, and O–C=O bonds, respectively, indicating the successful incorporation of a-FePO_4_ into the layered GO structure. In contrast, the C 1s spectra of a-FePO_4_-rGO-1 (Figure 3b) show three distinct peaks at 284.8 eV, 286.3 eV, and 288.3 eV, assigned to C–C/C=C, C=O, and O–C=O, respectively [28]. Compared with a-FePO_4_-GO, the peak intensities of C=O and C–O are significantly reduced, which can be attributed to the thermal reduction in GO to rGO during the high-temperature process, resulting in the removal of most oxygen-containing functional groups. However, the O–C=O peak remains present without a drastic decrease in intensity, suggesting that O–C=OH groups are relatively stable even under high-temperature treatment. These results demonstrate that XPS provides critical insights into the chemical changes during the reduction process, confirming the effective formation of rGO and the retention of certain functional groups influencing the composite’s properties.

### 3.2. Electrochemical Performance of the a-FePO_4_ and Its Composites

**Figure 4:**
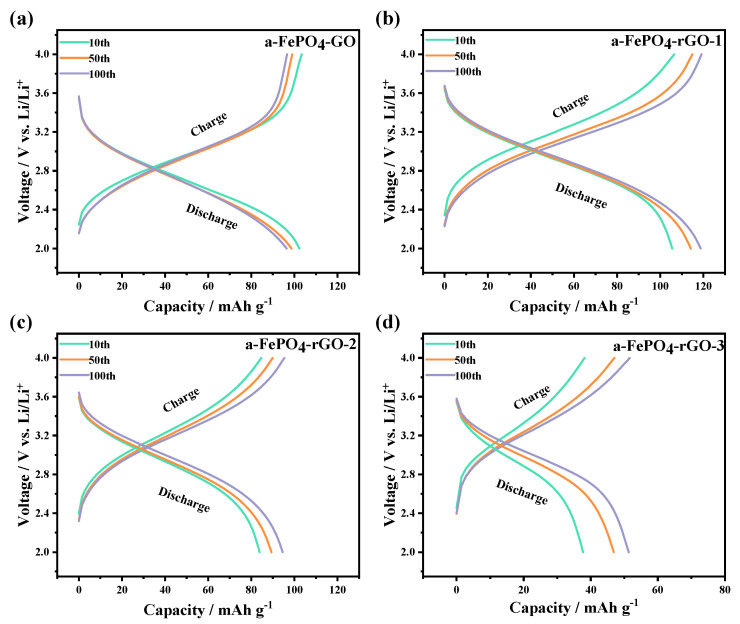
Charge–discharge curves for a-FePO_4_-GO and its high-temperature calcined composite electrodes at 1C rate. (**a**) a-FePO_4_-GO; (**b**) a-FePO_4_-rGO-1; (**c**) a-FePO_4_-rGO-2; (**d**) a-FePO_4_-rGO-3.

The charge–discharge curves of lithium-ion batteries reflect the thermodynamic and kinetic characteristics of their internal electrochemical reactions. Figure 4 displays the charge–discharge curves at the 10th, 50th, and 100th cycles for a-FePO_4_-GO and its high-temperature calcined composite electrodes, measured at a 1C rate. The results indicate that all four electrodes (a-FePO_4_-GO and a-FePO_4_-rGO-1/-2/-3) exhibit smooth, inclined charge–discharge profiles without distinct voltage plateaus and demonstrate a high overlapping degree among consecutive cycles. Among them, the a-FePO_4_-rGO-1 electrode (Figure 4b) delivered the best electrochemical performance, retaining a discharge specific capacity of 117 mAh·g^−1^ after 100 cycles. In comparison, the a-FePO_4_-GO electrode (Figure 4a) maintained a capacity of 95 mAh·g^−1^ under the same conditions. The superior performance of a-FePO_4_-rGO-1 is attributed to the reduction in GO to rGO during calcination at 250 °C under N_2_ atmosphere. The resulting rGO, exhibiting a structure analogous to single-layer graphene, significantly enhances lithium-ion conductivity. As shown in Figure 4c,d, the a-FePO_4_-rGO-2/-3 electrodes exhibited much lower discharge specific capacities of 95 mAh·g^−1^ and 50 mAh·g^−1^ after 100 cycles, respectively. Although all three types of a-FePO_4_-rGO composites remained amorphous in structure according to XRD, their electrochemical performance varied considerably with increasing calcination temperature. The degradation in charge–discharge behavior at higher temperatures may be due to a tendency of a-FePO_4_ to transform toward the crystalline phase, which is consistent with XRD observations. Higher temperatures promote the formation of crystalline FePO_4_, a more thermodynamically stable phase that hinders lithium-ion intercalation and deintercalation, leading to the rapid decline in capacity observed for a-FePO_4_-rGO-2 and a-FePO_4_-rGO-3. Furthermore, in contrast to the a-FePO_4_-GO electrode, the discharge specific capacities of the three a-FePO_4_-rGO electrodes gradually increased with cycling, indicating that the presence of rGO helps to stabilize and even enhance the cyclic performance of lithium-ion batteries [29].

**Figure 5:**
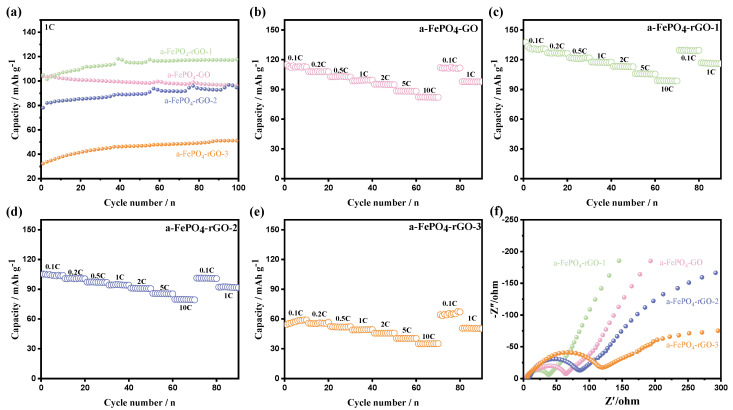
(**a**) Cyclic charge/discharge performance of a-FePO_4_-GO and a-FePO_4_-rGO-1/-2/-3; (**b**–**e**) Rate capability curves of a-FePO_4_-GO and a-FePO_4_-rGO-1/-2/-3 electrodes at various rates from 0.1 C to 10 C; (**f**) EIS plots of a-FePO_4_-GO and a-FePO_4_-rGO-1/-2/-3.

Figure 5a presents the charge/discharge cycling profiles of a-FePO_4_-GO composite electrodes calcined at different temperatures. The results demonstrate that the a-FePO_4_-rGO-1 electrode maintained a discharge specific capacity of 117 mAh·g^−1^ after 100 cycles at 1 C, whereas the uncalcined a-FePO_4_-GO electrode retained only 95 mAh·g^−1^ under the same testing conditions. This improvement can be attributed to the significantly enhanced ionic conductivity imparted by the presence of rGO. The a-FePO_4_-rGO-2/-3 electrodes exhibited lower discharge specific capacities of 95 mAh·g^−1^ and 52 mAh·g^−1^ after 100 cycles at 1 C, respectively, which is consistent with the charge/discharge performance mentioned before.

Figure 5b–e show the rate capability curves of the a-FePO_4_-GO-based electrodes calcined at different temperatures, measured at rates ranging from 0.1 C to 10 C. For all four composite materials (a-FePO_4_-GO and a-FePO_4_-rGO-1/-2/-3), the discharge specific capacity gradually decreased as the current rate increased. The relatively uniform decline in capacity across rates indicates stable electrochemical performance despite differences in absolute capacity values. The incorporation of GO not only enhanced the electrochemical properties of a-FePO_4_ but also contributed to the improved stability of the electrodes. As shown in Figure 5c, the a-FePO_4_-rGO-1 electrode exhibited the most promising rate performance, with discharge specific capacities of 130, 126, 121, 117, 113, 105, and 98 mAh·g^−1^ at rates of 0.1 C, 0.2 C, 0.5 C, 1 C, 2 C, 5 C, and 10 C, respectively. Under the same rates, the a-FePO_4_-GO electrode delivered capacities of 112, 107, 102, 98, 95, 88, and 82 mAh·g^−1^, while the a-FePO_4_-rGO-2/-3 electrodes showed progressively lower values of 104/57, 100/55, 97/51, 94/49, 90/45, 85/40, and 79/35 mAh·g^−1^, respectively. These results collectively demonstrate that moderate-temperature reduction in GO to rGO effectively enhances both the capacity and rate capability of a-FePO_4_-based electrodes, whereas excessively high calcination temperatures lead to structural ordering and performance degradation.

EIS is a widely used technique for characterizing the electrode/electrolyte interface by measuring frequency-dependent impedance, thereby revealing charge transfer kinetics, interfacial stability, and mass transport properties in electrochemical systems. Accordingly, we systematically characterized the impedance properties of a-FePO_4_-GO and a-FePO_4_-rGO-1/-2/-3 to comprehensively evaluate their interfacial evolution and stability during electrochemical operation. The EIS data of all materials (Figure 5f) exhibit similar features: a semicircle in the medium-to-high frequency region and a linear Warburg tail in the low-frequency region, corresponding to charge transfer and Li^+^ diffusion processes, respectively. Analysis of the impedance characteristics reveals notable differences in electrochemical behavior. Among them, a-FePO_4_-rGO-1 shows the smallest semicircle diameter and the steepest Warburg slope, indicating optimal charge transfer kinetics and lithium-ion diffusion efficiency. These favorable impedance properties are directly correlated with its superior electrochemical performance (~130 mAh·g^−1^ at 0.1 C). In contrast, a-FePO_4_-GO exhibits a larger semicircle diameter than a-FePO_4_-rGO-1, though its Warburg slope is similar, suggesting that the presence of rGO significantly reduces the interfacial resistance in a-FePO_4_-rGO-1. With further increase in calcination temperature, the semicircle diameters of a-FePO4-rGO-2 and a-FePO_4_-rGO-3 become progressively larger. Moreover, a-FePO_4_-rGO-3 displays the shallowest Warburg slope, indicating that elevated temperatures may promote the crystallization of a-FePO_4_, resulting in the deteriorating electrochemical activity of FePO_4_ and hindered Li^+^ (de)intercalation kinetics, thereby slowing overall charge transfer. The impedance parameters confirm that the composite formed between a-FePO_4_ and rGO at 250 °C constitutes the most effective conductive network, simultaneously enhancing both electronic and ionic transport.

## 4. Conclusions

In summary, this work successfully demonstrates the transformation of recycled quartz-type FePO_4_ into a-FePO_4_ and its subsequent enhancement through compositing with rGO. The thermal reduction temperature plays a pivotal role in determining the final structure and performance of the a-FePO_4_-rGO composites. While all composites remained amorphous below 450 °C, a tendency towards crystallization was observed with increasing temperature. Among the series of composites, a-FePO_4_-rGO-1 (250 °C) delivered the most outstanding overall performance, retaining a discharge capacity of 117 mAh·g^−1^ after 100 cycles at 1 C, which is significantly higher than that of a-FePO_4_-GO (95 mAh·g^−1^), a-FePO_4_-rGO-2 (95 mAh·g^−1^), and a-FePO_4_-rGO-3 (52 mAh·g^−1^). Similarly, its rate capability (98 mAh·g^−1^ at 10 C) surpassed all other composites, which exhibited rapidly decaying capacities under high current densities. EIS further confirmed that a-FePO_4_-rGO-1 possessed the smallest charge transfer resistance and the most favorable Li^+^ diffusion kinetics, underscoring the critical advantage of its optimized conductive network. In contrast, composites treated at higher temperatures showed increased resistance and sluggish kinetics, attributable to incipient crystallization and aggravated agglomeration. This study highlights that integrating recycled active materials with a rationally designed carbon matrix via a mild thermal treatment is a highly effective strategy for developing sustainable and high-performance cathode materials for advanced lithium-ion batteries.

## Data Availability

The original contributions presented in this study are included in the article/Supplementary Material. Further inquiries can be directed to the corresponding author.

## Supplementary Materials

The following supporting information can be downloaded at https://www.mdpi.com/article/10.3390/ma18214850/s1. Figure S1: The SEM images and corresponding magnified views of (a,b) a-FePO_4_-rGO-2; (c,d) a-FePO_4_-rGO-3; Figure S2: XPS survey of (a) a-FePO_4_; (b) a-FePO_4_-GO. (c) a-FePO_4_-rGO-1.

## Abbreviations

The following abbreviations are used in this manuscript:

SEM	Scanning Electron Microscopy
XRD	X-Ray Diffraction
XPS	X-Ray Photoelectron Spectroscopy
LFP	LiFePO_4_
LIBs	Lithium-Ion Batteries
FePO_4_	Amorphous FePO_4_
GO	Graphene Oxide
rGO	Reduced Graphene Oxide

## References

[1] Harper G., Sommerville R., Kendrick E., Driscoll L., Slater P., Stolkin R., Walton A., Christensen P., Heidrich O., Lambert S. (2019). Recycling lithium-ion batteries from electric vehicles. Nature.

[2] Lei S., Sun W., Yang Y. (2024). Comprehensive Technology for Recycling and Regenerating Materials from Spent Lithium Iron Phosphate Battery. Environ. Sci. Technol..

[3] Gangaja B., Nair S., Santhanagopalan D. (2021). Reuse, Recycle, and Regeneration of LiFePO_4_ Cathode from Spent Lithium-Ion Batteries for Rechargeable Lithium- and Sodium-Ion Batteries. ACS Sustain. Chem. Eng..

[4] Lv W., Wang Z., Cao H., Sun Y., Zhang Y., Sun Z. (2018). A Critical Review and Analysis on the Recycling of Spent Lithium-Ion Batteries. ACS Sustain. Chem. Eng..

[5] Zhang X., Zhu M. (2024). Recycling spent lithium-ion battery cathode: An overview. Green Chem..

[6] Chen X., Luo C., Zhang J., Kong J., Zhou T. (2015). Sustainable Recovery of Metals from Spent Lithium-Ion Batteries: A Green Process. ACS Sustain. Chem. Eng..

[7] Yang Y., Meng X., Cao H., Lin X., Liu C., Sun Y., Zhang Y., Sun Z. (2018). Selective recovery of lithium from spent lithium iron phosphate batteries: A sustainable process. Green Chem..

[8] Shentu H., Xiang B., Cheng Y.-J., Dong T., Gao J., Xia Y. (2021). A fast and efficient method for selective extraction of lithium from spent lithium iron phosphate battery. Environ. Technol. Innov..

[9] Dai Y., Xu Z., Hua D., Gu H., Wang N. (2020). Theoretical-molar Fe^3+^ recovering lithium from spent LiFePO_4_ batteries: An acid-free, efficient, and selective process. J. Hazard. Mater..

[10] Fang Y., Xiao L., Qian J., Ai X., Yang H., Cao Y. (2014). Mesoporous amorphous FePO_4_ nanospheres as high-performance cathode material for sodium-ion batteries. Nano Lett..

[11] Wang L., He X., Sun W., Wang J., Li Y., Fan S. (2012). Crystal orientation tuning of LiFePO_4_ nanoplates for high rate lithium battery cathode materials. Nano Lett..

[12] Wang Z., Lu Y. (2019). Facile Construction of High-Performance Amorphous FePO_4_/Carbon Nanomaterials as Cathodes of Lithium-Ion Batteries. ACS Appl. Mater. Interfaces.

[13] Yu J., Hu S., Zhang Y., Liu Y., Ren W., Zhu A., Feng Y., Wang Z., Rao D., Yang Y. (2025). Graphene-Enhanced FePO_4_ Composites with Superior Electrochemical Performance for Lithium-Ion Batteries. Materials.

[14] Wang W., Gao P., Zhang S., Zhang J. (2017). A cylindrical FePO4/MWCNTs composite with a 3D conductive network structure used as a cathode material for lithium-ion batteries. J. Alloys Compd..

[15] Jiang Y., Song D., Wu J., Wang Z., Huang S., Xu Y., Chen Z., Zhao B., Zhang J. (2019). Sandwich-like SnS_2_/Graphene/SnS_2_ with Expanded Interlayer Distance as High-Rate Lithium/Sodium-Ion Battery Anode Materials. ACS Nano.

[16] Zhu Y., Tang S., Shi H., Hu H. (2014). Synthesis of FePO_4_·xH_2_O for fabricating submicrometer structured LiFePO_4_/C by a co-precipitation method. Ceram. Int..

[17] Zhao X., Luo M., Peng K., Zhang Z., Cheng B., Wang B., Zhu C., Yan X., Shi K. (2021). Low-Temperature Synthesis of Amorphous FePO_4_@rGO Composites for Cost-Effective Sodium-Ion Batteries. ACS Appl. Mater. Interfaces.

[18] Zeng S., Xu Q., Jin H., Zeng L., Wang Y., Lai W., Yao Q., Zhang J., Chen Q., Qian Q. (2022). A green strategy towards fabricating FePO_4_-graphene oxide for high-performance cathode of lithium/sodium-ion batteries recovered from spent batteries. J. Electroanal. Chem..

[19] Xu W., Yao J., Liu Z., Jiang J., Xiao S., Li Y. (2025). Amorphous porous FePO_4_/reduced graphene oxide nanocomposite cathode material prepared from the leaching solution of jarosite residue with excellent lithium/sodium storage performance. J. Power Sources.

[20] Liu Y., Xu S., Zhang S., Zhang J., Fan J., Zhou Y. (2015). Direct growth of FePO_4_/reduced graphene oxide nanosheet composites for the sodium-ion battery. J. Mater. Chem. A.

[21] Chu S., Guo S., Zhou H. (2021). Advanced cobalt-free cathode materials for sodium-ion batteries. Chem. Soc. Rev..

[22] Wang H., Zhang S., Cao H., Liu Y., Zhou L., Yin Q. (2026). Controllable synthesis of spherical FePO_4_•2H_2_O: Insights into crystal-face interactions and amorphous-to-crystalline transition. Chem. Eng. Sci..

[23] Politano G.G., Versace C. (2022). Electrical and Optical Characterization of Graphene Oxide and Reduced Graphene Oxide Thin Films. Crystals.

[24] Amiri H., Nikookhesal A., Murugan D., Scholz S., Frentzen M., Cao Y., Nickl P., Radnik J., Stockmann J.M., Vu X.-T. (2025). High precision correlative analysis of dielectric behavior evolution and anisotropy in graphene oxide thin film as a function of thermal annealing parameters. Nano Trends.

[25] Chen M., Zheng Z., Wang Q., Zhang Y., Ma X., Shen C., Xu D., Liu J., Liu Y., Gionet P. (2019). Closed Loop Recycling of Electric Vehicle Batteries to Enable Ultra-high Quality Cathode Powder. Sci. Rep..

[26] Qin X., Zhang H., Wang Z., Jin Y. (2018). Magnetic chitosan/graphene oxide composite loaded with novel photosensitizer for enhanced photodynamic therapy. RSC Adv..

[27] Zhang X., Sun Q., Liang X., Gu P., Hu Z., Yang X., Liu M., Sun Z., Huang J., Wu G. (2024). Stretchable and negative-Poisson-ratio porous metamaterials. Nat. Commun..

[28] Zhang Y., Li H., Zhang Q., Guo X., Zhu Z. (2024). Impact of ultrasonic treatment duration on the microstructure and electrochemical performance of NiZnCo_2_O_4_ electrode materials for supercapacitors. Sci. Rep..

[29] Wang L., Wang D., Dong Z., Zhang F., Jin J. (2013). Interface chemistry engineering for stable cycling of reduced GO/SnO_2_ nanocomposites for lithium ion battery. Nano Lett..

