# Supplementary material for: Optimization of Recycled a-FePO4/rGO Composites via Thermal Reduction for Enhanced-Performance Lithium-Ion Batteries
Source: Materials (Basel). 2025 Oct 23;18(21):4850. doi: 10.3390/ma18214850 (PMC12610454; doi:10.3390/ma18214850)
Supplement: Supplementary file 1 [file materials-18-04850-s001.zip › materials-3910928-supplementary.pdf]

# Supplementary Information

# Optimization of Recycled a-FePO4/rGO Composites via Thermal Reduction for Enhanced-Performance Lithium-Ion Batteries

Shuchun Hu[1], Jinde Yu[1]*, Hua Chen[2], Zengbin Lin[3], Fengchun Zhang[1], Meiling Guo[1], Aipeng Zhu[1], Yin Liu[1]

[1] Yibin Research Institute & School of Materials and Environmental Engineering, Chengdu Technological Uni-versity, Chengdu 611730, China.

[2] Sichuan Contemporary Amperex Technology Co., Ltd., Yibin 644005, China.

[3] Contemporary Amperex Technology Co., Ltd. (CATL), Ningde 352100, China.

**Keywords**: Lithium-ion batteries; Spent $LiFePO_4$ recycling; Amorphous $FePO_4$/rGO composites;

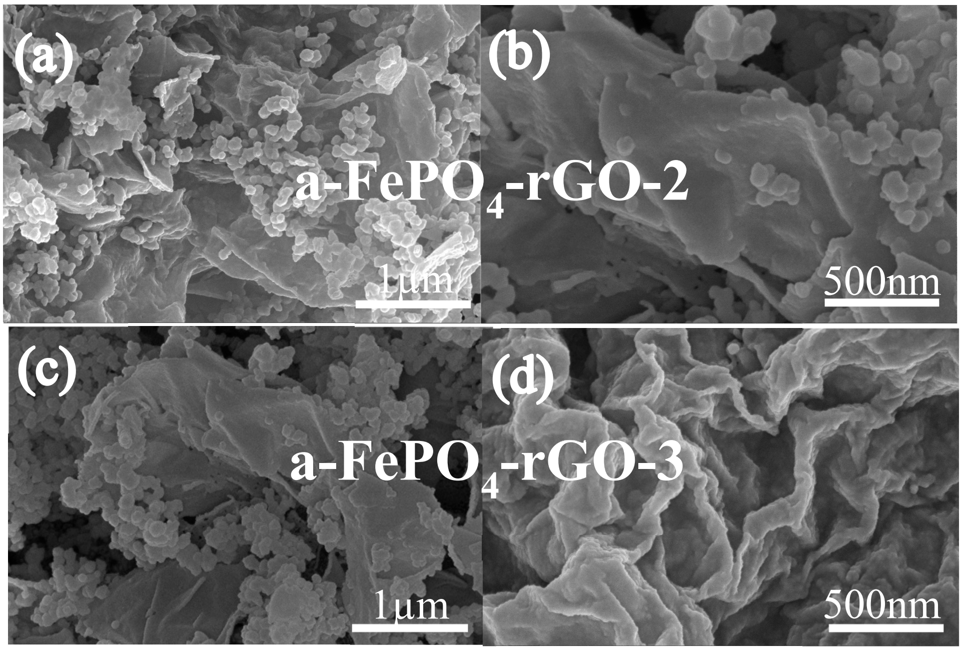


**Figure S1** The SEM images and corresponding magnified views of (a-b) a-$FePO_4$-rGO-2; (c-d) a-$FePO_4$-rGO-3

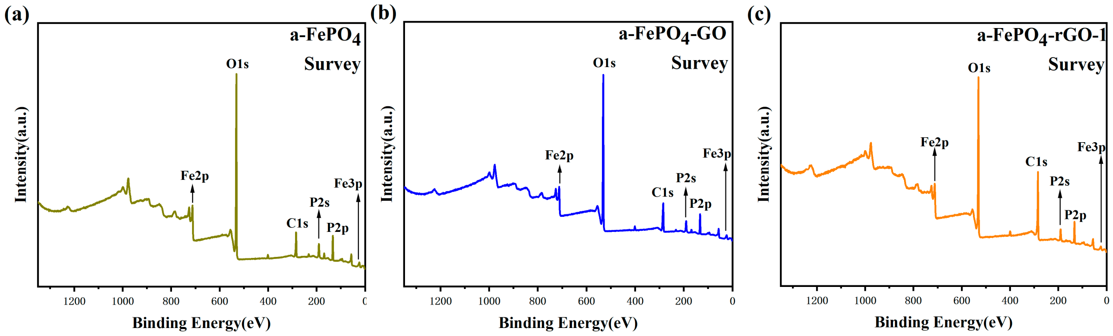


**Figure S2** XPS survey of (a) a-$FePO_4$; (b) a-$FePO_4$-GO. (c) a-$FePO_4$-rGO-1.
